# Optimum Mix of Tunneling Coal Gangue as a Highway Base Material Through Delphi–Entropy Weight–TOPSIS and Microstructure Analysis

**DOI:** 10.3390/ma18102191

**Published:** 2025-05-09

**Authors:** Decai Wang, Baiyu Wang, Zongyuan Wu, Jiawei Wei, Riran Wang, Jingjiang Wu, Shenzhen Ding

**Affiliations:** 1 School of Civil Engineering and Communication, North China University of Water Resources and Electric Power, Zhengzhou 450045, China; wangdecai@ncwu.edu.cn (D.W.); wangbaiyu68@163.com (B.W.); weijw0820@126.com (J.W.); dingshenzhen@ncwu.edu.cn (S.D.); 2 School of Water Conservancy and Transportation, Zhengzhou University, 100 Science Rd., Zhengzhou 450001, China; wangrr@zzu.edu.cn; 3 China Construction Seventh Engineering Division Corporation Ltd., Zhengzhou 450004, China; wujingjiang@cscec.com

**Keywords:** solid waste utilization, tunneling coal gangue, comprehensive properties, quantitative evaluation, improved TOPSIS model, internal mechanisms

## Abstract

Using coal gangue in highway base construction provides a sustainable and high-value solid waste recycling approach. This research focused on the mechanical and durability properties of coal gangue from tunneling operations. Six experimental tests, such as unconfined compressive strength (UCS), flexural–tensile strength (FTS), etc., were carried out. The impact of aggregate gradation on coal gangue mixtures’ performance was systematically evaluated. XRD and SEM were used to explore the microstructural mechanisms in cement-stabilized coal gangue–gravel mixtures (CGM). An improved evaluation model, the Delphi–entropy weight–TOPSIS (DET) method, integrating Delphi and entropy weighting, was proposed. Together with an advanced radar chart, it evaluates eight performance criteria, including mechanical, durability, economic, and environmental aspects. The results show that increasing the coal gangue content in mixtures decreases UCS, dynamic compressive rebound modulus (DCRM), FTS, fatigue life, and drying shrinkage performance. Coarse aggregates relieve drying shrinkage, while fine ones improve long-term mechanical properties. Gradation T1~3 promotes the formation of C–S–H gel and AFt crystals, enhancing compactness. Based on the DET model’s quantitative evaluation, T1~3 was determined as the optimal mix for expressway bases, achieving a balance between mechanical performance, durability, and sustainability.

## 1. Introduction

Coal gangue, an inevitable byproduct of coal mining and washing, accounts for about 15% of the total raw coal output [1]. With an estimated accumulation exceeding 7 billion tons, covering over 200,000 hectares, coal gangue is one of the largest and most environmentally damaging solid wastes in China [2,3,4]. Its vast accumulation leads to the consumption of valuable land resources and poses significant environmental risks, such as the potential for collapse, surface subsidence, and spontaneous combustion. Moreover, the leaching of heavy metals from coal gangue can severely contaminate surrounding soil and groundwater ecosystems [5]. Nedeljković et al. [6] investigated alkali-activated fly ash and slag pastes, revealing that higher slag content improved compressive strength but increased autogenous shrinkage, highlighting the complexity of optimizing binder ratios for such materials. Moghadam et al. [4] reviewed the durability of geopolymers using industrial wastes such as coal gangue, emphasizing their resistance to acid corrosion and high temperatures, which underscores their potential in harsh environments. Oliveira et al. [7] noted that alkali-activated materials (AAMs), including geopolymers, can utilize industrial byproducts such as fly ash and blast furnace slag as sustainable alternatives to Portland cement. Their work highlighted the influence of reaction kinetics, activator types, and curing conditions on geopolymer durability under environmental challenges such as carbonation and sulfate attack. Therefore, effective utilization and high-value applications of coal gangue are crucial for reducing its environmental impact and promoting sustainability.

In response to the spatial and environmental challenges associated with coal gangue, research has focused on exploring its material properties and potential applications, aiming to develop innovative strategies for its repurposing. Several uses, including power generation, ceramic brick production, cement manufacturing, and as an engineering fill material, have made significant progress in improving coal gangue’s resource efficiency. From a highway engineering perspective, the angularity and particle size distribution of coal gangue make it a promising partial substitute for conventional road construction materials, especially given the growing scarcity and high cost of natural sand and gravel. Huseien et al. [8] demonstrated that incorporating fly ash into alkali-activated concretes reduced drying shrinkage and enhanced acid resistance, showcasing the benefits of blended binders for durable pavement applications. Mischinenko et al. [9] explored microwave foaming of coal gangue-based alkali-activated materials, optimizing parameters to achieve uniform pore structures and improved thermal insulation, offering energy-efficient processing alternatives. Priyadharshini et al. [10] investigated the use of excavated soils with varying plasticity as fine aggregates in fly ash-based geopolymer mortars, revealing that soil type, NaOH molarity, and curing temperature significantly impact workability, strength, and shrinkage. Their study showed that even high-plasticity soils can be effectively utilized through geopolymerization, providing insights into sustainable aggregate substitution in construction. Consequently, using coal gangue in road construction presents a viable solution for managing industrial solid waste [11,12]. Since the 1980s, much of China’s research has focused on applying coal gangue as a subbase material and roadbed reinforcement, leveraging its aggregate strength and pozzolanic reactivity. These efforts have yielded considerable knowledge and led to the maturation of technologies for low-grade road construction [13].

Over time, the scope of coal gangue applications has expanded to include roadbed filling, hydraulic embankments, and foundation layers [14,15]. However, its high-value use remains limited due to challenges such as compositional variability, the risk of spontaneous combustion from residual coal, and the softening or decomposition of wet coal gangue, which compromise its load-bearing capacity and structural integrity. The chemical composition and mineralogical properties of coal gangue can vary widely based on its origin, production process, and treatment methods [16,17]. Frasson et al. [18] highlighted the influence of particle size and activation methods on the mechanical behavior of coal gangue in concrete, emphasizing the need for standardized processing to ensure consistent performance. To better understand and enhance its suitability for highway engineering, researchers have studied its performance under various conditions. For example, Gao et al. [19] found that increasing coal gangue replacement rates negatively affected the compressive strength and elastic modulus of concrete. Similarly, Guan et al. [20] demonstrated that cement-stabilized coal gangue exhibits lower dry density and moisture content than gravel but still provides adequate strength for use in secondary roads. Cao et al. [21] compared the properties of cement, lime, and fly ash-stabilized coal gangue mixtures, concluding that inorganic binder-stabilized coal gangue is suitable for tertiary road subbase applications. Zhou et al. [22] developed models to predict the stress–strain behavior of coal gangue concrete, and Guan et al. [23] found that freeze–thaw cycles negatively affected the compressive performance of coal gangue concrete. Research by Li et al. [24] showed that coal gangue’s higher porosity and water absorption contribute to better bonding with cement, though coal gangue mixtures often exhibit lower mechanical strength due to extensive microporosity. Gao et al. [25] observed that increasing the coal gangue replacement rate reduces both the mechanical strength and durability of mixtures, making them unsuitable for express highways. In contrast, Zhu et al. [26] showed that coal gangue as a coarse aggregate can improve the workability of concrete, while using coal gangue powder as a supplementary cementitious material enhances late-stage strength. Yu et al. [27] found that replacing natural aggregates with coal gangue reduces the mechanical strength of mixtures, but the mixtures still meet regulatory standards. Furthermore, over time, the microstructure of coal gangue mixtures densifies, resulting in improved strength. In conclusion, while coal gangue has been validated for use in low-grade road bases, its mechanical and durability shortcomings, such as reduced strength and freeze–thaw resistance, restrict its applicability in high-grade road construction.

Despite the advancements, current research on the comprehensive utilization of coal gangue in road base layers still faces several gaps, particularly in the context of its use in high-grade subbase materials. Key issues are provided below. (1) Most studies have focused on the experimental application of coal gangue in low-grade road bases and subbases, with limited systematic research on its mechanical properties, durability, and performance mechanisms, especially for high-grade road base materials, resulting in insufficient data for effective application in highway and railway base structures. (2) There is a notable lack of comparative research on the effects of different replacement methods on the mechanical properties and durability of coal gangue mixtures. (3) The physicochemical properties of coal gangue vary significantly due to factors such as geographical origin, production processes, and treatment methods, leading to inconsistencies in particle size and strength, even from the same production site. Therefore, further investigation into the comprehensive performance and underlying mechanisms of cement-stabilized coal gangue mixtures is of significant engineering value for their application in road base layers.

Although there are still gaps in the research, the aforementioned studies highlight the crucial role of coal gangue’s replacement rate and blending method in determining the overall performance of cement-stabilized base mixtures. However, there is a lack of systematic methods to comprehensively evaluate the performance of coal gangue mixtures. Thus, the development of a quantitative evaluation model for assessing the comprehensive performance of coal gangue mixtures is essential to identify the optimal blending strategy. Modern computational techniques, including grey theory, fuzzy mathematics, neural networks, TOPSIS, entropy weighting methods, analytic hierarchy processes, and Delphi methods, have been increasingly applied to the comprehensive evaluation of pavement performance. For example, Vidhi et al. [28] developed an artificial neural network model for rapid pavement condition evaluation, while Xu et al. [29] used fuzzy hierarchical evaluation methods to assess the comprehensive utilization of steel slag. Wu et al. [30] proposed an evaluation system for green building design, and Luo et al. [31] developed a multi-criteria decision-making approach for assessing urban solid waste management strategies. While methods such as Delphi and hierarchical analysis are useful for engineering risk evaluation, their subjective weighting can introduce biases, whereas objective weighting methods offer more theoretical support but may not align perfectly with real-world importance. The TOPSIS method, which evaluates alternatives based on positive and negative ideal solutions, normalizes data to account for different dimensions and provides a more accurate reflection of alternative performance, making it increasingly applicable in various engineering fields [32].

Although mathematical models are widely used in highway engineering, studies applying these models to evaluate the performance of coal gangue mixtures are still rare. Given the complex and multidimensional nature of road performance, previous research often relied on singular mathematical methods, which may have led to biased results. To address these issues, this study proposed a DET evaluation model that combined both subjective and objective methods to determine the relative importance of various performance indicators and employed the TOPSIS method to establish a comprehensive evaluation model for coal gangue mixtures. This approach mitigated the limitations of singular methods and provided a more balanced and accurate evaluation. The TOPSIS method was particularly suitable for this study as it does not impose strict sample size or objective function constraints, making it ideal for capturing the multifactorial impact on performance.

The primary goal of this research was to recommend optimal mix designs for high-grade road subbases, extend pavement lifespan, and promote the high-value utilization of coal gangue in highway engineering projects. The study focused on coal gangue sourced from tunneling operations in northern Henan Province, conducting laboratory experiments to assess its physicochemical properties. The mechanical properties of coal gangue mixtures were tested using UCS, FTS, and DCRM tests. Durability was evaluated through freeze–thaw, drying shrinkage, and fatigue tests. Microstructural analysis, including XRD and SEM, was conducted to examine the mechanisms underlying the performance of these mixtures. A DET evaluation model was proposed to determine the weighting of performance indicators and evaluate the mixtures across various aspects. Finally, the results were visualized using an advanced radar chart method, where the sector angles were determined based on the combined Delphi–entropy weights.

## 2. Materials and Methods

### 2.1. Materials

#### 2.1.1. Coal Gangue

**Figure 1:**
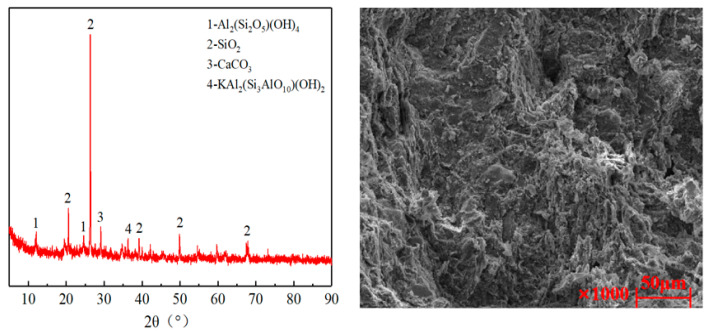
Coal gangue and its microscopic test results.

Coal gangue (Hebi City, China) has a gray or gray–black granular appearance. Upon crushing, it is classified into four distinct particle size ranges: 0–4.75 mm, 4.75–9.5 mm, 9.5–19 mm, and 19–31.5 mm. To analyze the chemical composition and microstructure of coal gangue, XRD and SEM were performed, as illustrated in Figure 1. The primary mineral constituents of coal gangue included quartz, kaolinite, and illite, which featured a flaky or layered structure, interspersed with porous cavities. This structural arrangement resulted in a relatively loose configuration, which may have contributed to its higher water absorption capacity and lower mechanical strength. The technical specifications of coal gangue are provided in Table 1.

Table 2 presents the chemical composition of coal gangue, which primarily included SiO_2_, Al_2_O_3_, CaO, and Fe_2_O_3_. Notably, the contents of SiO_2_ and Al_2_O_3_ were relatively high, measuring 47.02% and 21.37%, respectively.

#### 2.1.2. Aggregate

**Figure 2:**
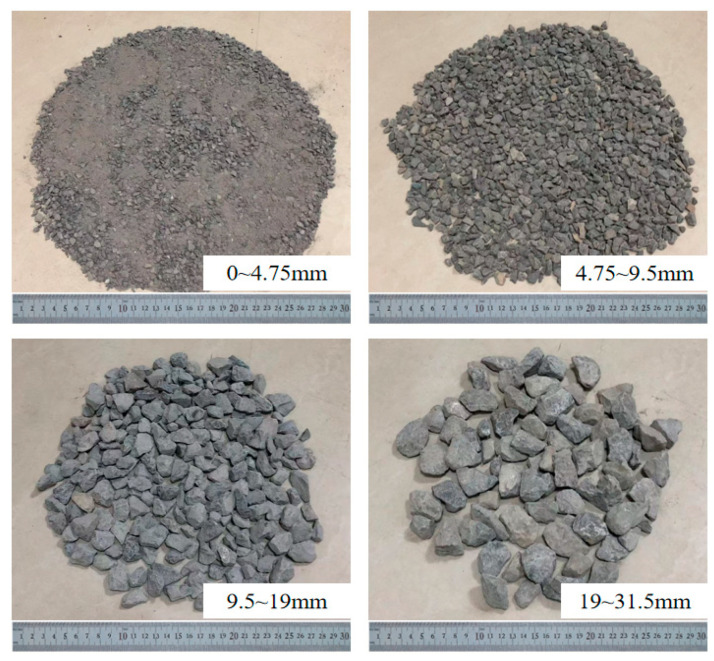
Limestone aggregate.

The experiment utilized a limestone aggregate, which was crushed and classified into four size ranges consistent with coal gangue, as shown in Figure 2. The technical specifications for each size fraction of the aggregate were tested, and the results are presented in Table 3.

Comparing Table 1 and Table 3, it can be observed that coal gangue had a lower apparent density and a higher water absorption, crushing value, and flakiness content compared to the limestone aggregate.

#### 2.1.3. Cement and Water

The cement used in the experiment was ordinary Portland cement PO 42.5 produced by Henan Tongli Cement Co., Ltd. (Zhengzhou, China). Following the testing methods outlined in the *Specifications for Testing Cement and Cement Concrete in Highway Engineering* (JTG 3420-2020), various technical specifications of cement were evaluated, with the results shown in Table 4. The water used in the experiments was potable water.

### 2.2. Gradation Design

**Figure 3:**
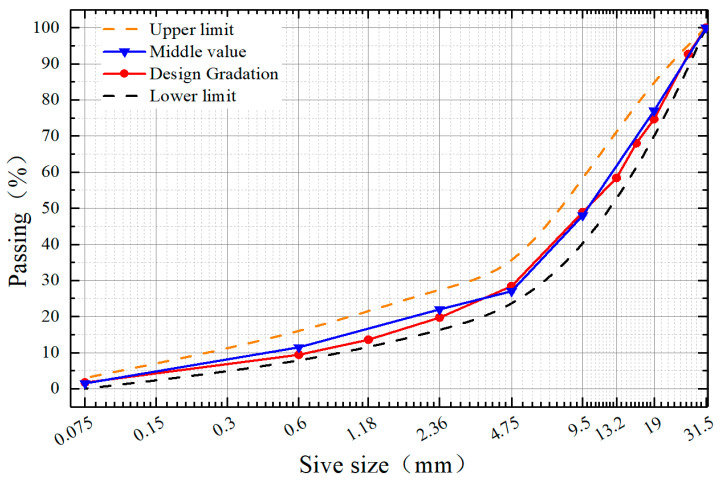
Gradation design curve.

The experiment utilized the C-B-3 dense-graded aggregate recommended in the *Technical Specifications for the Construction of Highway Pavement Base Courses* (JTG/T F20-2015). Through optimization calculations, the optimal mixing ratio for the four size fractions of the coal gangue aggregate was determined to be as follows: 19~31.5 mm: 9.5~19 mm:4.75~9.5 mm:0~4.75 mm = 27%:25%:20%:28%. The corresponding grading curve and sieve passage rates are shown in Figure 3 and Table 5, respectively.

Two mixing methods were employed: proportion substitution and particle size substitution. In the proportion substitution method, coal gangue was completely substituted for crushed stone according to the grading, with replacement ratios set at 20%, 40%, 60%, and 80%. This approach aimed to assess the impact of varying amounts of crushed stone on the technical performance of the mixture. In the particle size substitution method, a specific size fraction of coal gangue was replaced by gravel, allowing for an evaluation of the differential performance between coarse and fine aggregates. The cement content was maintained at 4%, and the ten mixing schemes are summarized in Table 6.

### 2.3. Testing Methods

#### 2.3.1. The UCS Test

The maximum dry density and optimum moisture content of the CGM were determined using the Class C compaction test method specified in the *Specifications for Testing Inorganic-Bonded Stabilized Materials in Highway Engineering* (JTG E51-2009). This study analyzed the UCS test results at 7, 28, and 90 days to evaluate and compare the performance of eight experimental groups, aiming to identify the optimal mixing method.

#### 2.3.2. The FTS Test

**Figure 4:**
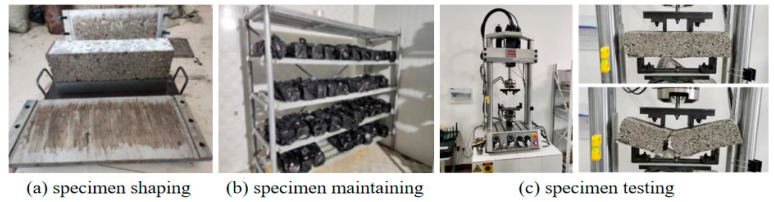
Flexural–tensile strength (FTS) test.

In accordance with the specimen preparation guidelines in JTG E51, beam specimens measuring 400 mm × 100 mm × 100 mm were fabricated. The central beam specimens underwent standard curing for 28 and 90 days, with saturation treatment applied 1 day before the end of the curing period. After curing, the specimens were placed in a universal testing machine for FTS testing, as illustrated in Figure 4. The test was performed under the displacement control mode using a four-point bending setup, with a loading rate of 50 mm/min. The ultimate load (P) at failure for each beam was recorded, and the average value was used as the final test result. The FTS of the mixture was then calculated using Equation (1).

(1) $$R_{S}=\frac{PL}{b^{2}h}$$

where $R_{s}$ represents the FTS (MPa); P represents the ultimate load at the time of damage to the specimen (kN); L is the length of the span, the distance between the two support points (mm); b represents the width of the specimen (mm); and h represents the height of the specimen (mm).

#### 2.3.3. The DCRM Test

**Figure 5:**
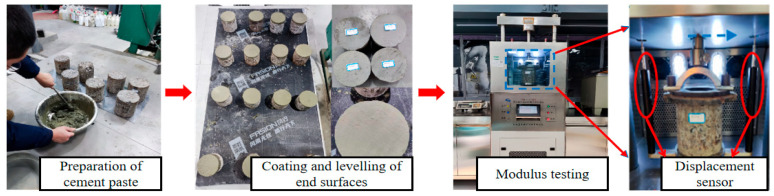
Dynamic compressive rebound modulus (DCRM) test.

In accordance with the specimen preparation guidelines in JTG E51, cylindrical specimens with dimensions of 150 mm × 150 mm were fabricated. These specimens were subjected to standard curing for 7, 28, and 90 days. After the curing period, the two ends of the cylinders were smoothed using cement paste and then treated with saturation for 1 day. Testing was conducted using an electric hydraulic testing machine, as shown in Figure 5. The experiment utilized a haversine load waveform with a frequency of 10 Hz, an intermittent period of 1 min, and six load levels ranging from 0.1 P to 0.6 P, with each load level applied 200 times. The DCRM was calculated using Equation (2).

(2) $$E_{dc}=\frac{\sigma }{\varepsilon }=\frac{F_{o}\times h}{\left(l_{o}-c\right)\times A}$$

where $E_{dc}$ represents the DCRM of the specimen (MPa); $F_{0}$ represents the load amplitude (N); $l_{o}$ represents the deformation amplitude (mm); c represents the corrected deformation amplitude (mm); h represents the height of the specimen (mm); and A represents the cross-sectional area of the specimen (mm^2^).

#### 2.3.4. The Freeze–Thaw Test

The freeze–thaw test was conducted using cylindrical specimens with dimensions of 150 mm × 150 mm. The UCS of the specimens was measured after five freeze–thaw cycles, following 28 days of curing. The freeze–thaw procedure was carried out as described below. Initially, the baseline mass m_0_ and the baseline UCS of the specimens were recorded after standard curing for 28 days. The specimens were then subjected to freezing at a constant temperature of −18 °C for 16 h, followed by immersion in water at 20 °C for 8 h to facilitate thawing. After removing the surface water, the specimens’ masses were recorded, and the process was repeated five times. Finally, the UCS of the specimens was tested. The freeze–thaw residual strength ratio was used to assess the frost resistance of the mixture. The relevant calculation formulas are provided in Equations (3) and (4).

(3) $$BDR=\frac{R_{DC}}{R_{C}}\times 100\%$$

(4) $$W=\frac{m_{0}-m_{5}}{m_{0}}\times 100\%$$

where *BDR* represents the freeze–thaw residual strength ratio (%); R_DC_ represents the UCS of the specimen after five freeze–thaw cycles (MPa); R_C_ represents the baseline UCS (MPa); W represents the mass loss rate due to freeze–thaw cycles (%); m_0_ represents the baseline mass (g); and m_5_ represents the mass of the specimen after five freeze–thaw cycles (g).

#### 2.3.5. Dry Shrinkage Test

**Figure 6:**
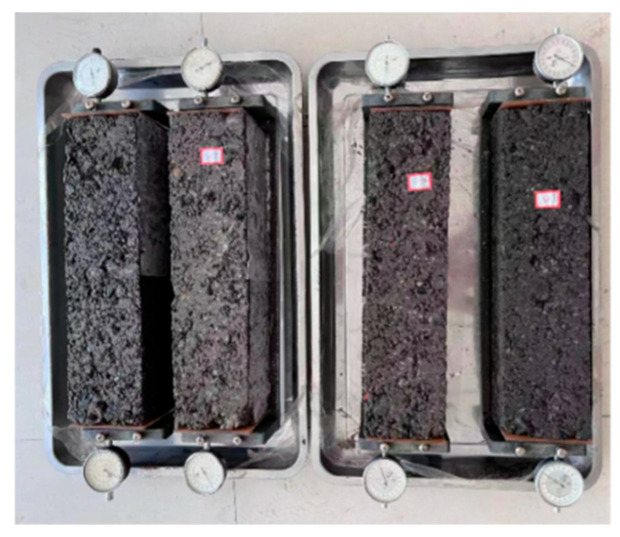
Drying shrinkage test.

The dry shrinkage test was carried out following the procedure outlined in JTG E51. The test specimens were beam-shaped, with dimensions of 100 mm × 100 mm × 400 mm. After 7 days of curing, the specimens were removed, and their surfaces were dried. Glass slides were attached to both ends, and the specimens were positioned on a shrinkage gauge, where they were secured, and the dial gauge was calibrated. The specimens were then placed in a dry shrinkage testing chamber, as illustrated in Figure 6. During the first week of the test, the mass of the specimens was measured daily to monitor water loss, while the corresponding dry shrinkage readings were recorded from the dial gauge. After the initial week, measurements were taken every two days, completing a total of 29 days for the dry shrinkage cycle. At the end of the test, the specimens were dried and weighed. The dry shrinkage performance was evaluated based on the water loss rate and dry shrinkage strain, with calculations performed using Equations (5)–(7).

(5) $$w_{i}=\frac{m_{i}-m_{i+1}}{m_{p}}$$

(6) $$\delta_{i}=\sum_{j=1}^{2}X_{i,j}-\sum_{j=1}^{2}X_{i+1,j}$$

(7) $$\varepsilon_{i}=\frac{\delta_{i}}{l}$$

#### 2.3.6. Fatigue Test

**Figure 7:**
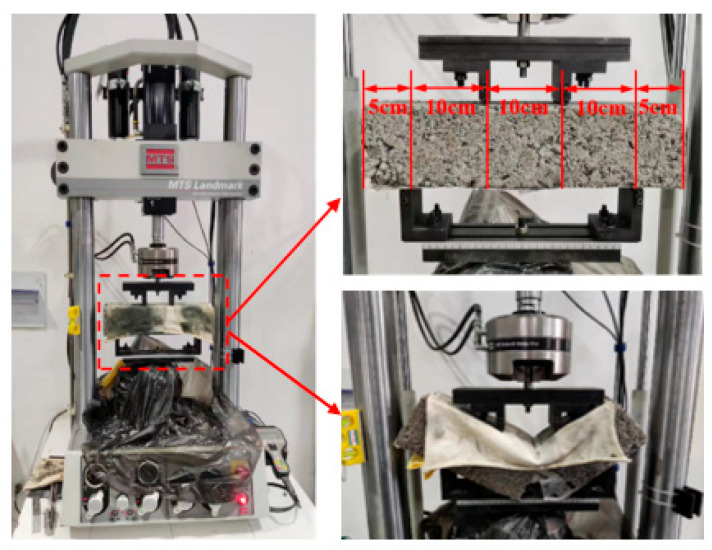
Fatigue test.

Fatigue tests were performed using an MTS 370.02 universal testing machine (Eden Prairie, MN, USA), with specimens identical to those used in the FTS tests. A four-point bending setup was utilized, and the tests were conducted under the stress control mode, subjecting the specimens to continuous sinusoidal loading at a frequency of 10 Hz. Four stress ratio levels (0.65, 0.70, 0.75, and 0.80) were applied to each set of specimens, with four replicates tested at each stress ratio. The maximum load was determined based on the FTS results of the specimen and the selected stress ratio, while the minimum load was set at 0.02 times the maximum load. Fatigue failure of the specimen was used as the criterion for assessing the fatigue performance of the mixture. The testing procedure is depicted in Figure 7.

#### 2.3.7. Microscopic Test

To prepare the XRD test samples, representative samples were selected, finely ground, and immersed in anhydrous ethanol for 1 d to halt hydration. The samples were then placed in a drying oven at 60 °C, sieved through a 0.075 mm mesh, and subsequently tested using a Bruker D8 XRD instrument (Billerica, MA, USA). The testing parameters were set as follows: Cu-Kα target, voltage of 40 kV, scanning rate of 5°/min, with a 2θ angle range of 5° to 90°.

For the SEM testing, the interface between the cement paste and the coal gangue aggregate was selected. The sample was immersed in anhydrous ethanol for 1 d to terminate the hydration reaction. It was then prepared into a specimen of approximately 1 cm^3^, gold-coated, and dried before being examined using a FEI Nova Nano 450 scanning electron microscope (Waltham, MA, USA).

### 2.4. DET Evaluation Model

The comprehensive evaluation of the performance of CGMs is essential in road engineering, as it directly impacts the stability and cost efficiency of road bases. However, assessing the performance of these mixtures is a complex process due to the numerous indicators and factors involved, as well as the diverse criteria employed by different stakeholders.

To tackle this challenge, this paper proposes a DET evaluation model to assess the overall performance of CGMs. The model addresses conflicts arising from varying evaluation criteria by integrating both subjective and objective weighting methods. Specifically, the Delphi method and the entropy weight method are combined to determine the weights for each performance indicator. Subsequently, a TOPSIS evaluation framework is established to comprehensively assess eight performance indicators, including mechanical properties, durability, economic viability, and environmental impact.

The combined weighting method, grounded in game theory, aims to balance conflicting weights derived from subjective and objective methods by using Nash equilibrium as the coordination objective [38]. This approach ensures a harmonious integration of subjective and objective weights, enabling a thorough incorporation of intrinsic information within the indicators. By reducing arbitrariness in weight assignment, it enhances the scientific rigor and rationality of the weighting process.

Further strengthening the evaluation, the TOPSIS method ranks samples based on their proximity to an ideal solution [39,40]. This process involves identifying both the optimal and worst solutions within a standardized, dimensionless data matrix, then calculating the distances of evaluation objects from these extremes. These distances form the basis for determining sample quality.

The integration of the combined weighting method with the TOPSIS framework enables the DET model to leverage the strengths of both approaches. First, it applies the combined weighting method to assign indicator weights, effectively integrating both subjective assessments and objective data. It then uses a hierarchical TOPSIS method to evaluate the performance of each scheme. This integrated approach overcomes the limitations of traditional single-method weighting techniques, maximizes the use of original data, and allows for a multidimensional evaluation of the dataset. Additionally, it combines localized and holistic assessments, offering a robust and comprehensive evaluation framework [41,42].

#### 2.4.1. Objective Weight Calculation Using the Entropy Weight Method

The entropy weight method primarily determines the weight coefficients of indicators by calculating the information entropy of each indicator, based on the degree of relative variation of the indicators. Indicators with a greater degree of relative variation are assigned higher weights. For a given evaluation object $M=\left(M_{1},\;M_{2},\ldots M_{m}\right)$, evaluation indicators $N=\left(N_{1},\;N_{2}\;,\ldots N_{n}\right)$, and evaluation values for object $M_{i}$ denoted as $N_{j}$, a matrix is formed, $r_{ij}(i=1,2,\ldots ,m;j=1,2,\ldots ,n)$, where $R={\left(r_{ij}\right)}_{m\times n}$ represents the evaluation value of the jth item under the ith indicator. The calculation steps for assigning weights using the entropy weight method are as follows:

(1) Standardization Processing.

To address the differences in dimensional units among the evaluation indicators, each indicator is dimensionlessly processed according to Equation (8).

(8) $$r_{ij}\prime =\frac{r_{ij}-\min\left(r_{1j},r_{2j},\ldots ,r_{mj}\right)}{\max\left(r_{1j},r_{2j},\ldots ,r_{mj}\right)-\min\left(r_{1j},r_{2j},\ldots ,r_{mj}\right)}$$

(2) Calculation of information entropy for each indicator.

The proportion $y_{ij}$ of the ith evaluation object under the jth indicator is calculated according to Equation (9):

(9) $$y_{ij}=\frac{r_{ij}\prime }{\sum_{i=1}^{m}r_{ij}\prime },(0\leq y_{ij}\leq 1)$$

(3) Calculation of the indicator’s information entropy value e, information effect value d, and evaluation indicator weights.

The information entropy value of the jth indicator is calculated using Equations (10) and (11):

(10) $$e_{j}=-K\sum_{i=1}^{m}y_{ij}\ln y_{ij}$$

(11) $$K=\frac{1}{\ln m}$$

The information effect value of the indicator is calculated using Equation (12):

(12) $$d_{j}=1-e_{j}$$

The weights of the evaluation indicators are calculated using Equation (13):

(13) $$v_{j}=\frac{d_{j}}{\sum_{j=1}^{n}d_{j}}$$

#### 2.4.2. Subjective Weight Calculation Using the Delphi Method

The Delphi method, also known as the expert scoring method, is a subjective evaluation technique that aims to achieve the most accurate results through subjective analysis. The specific steps are described below. First, invite $L\left(L\geq 2\right)$ experts to rank five evaluation indicators according to their understanding, thereby defining the expert set as $P=\left\{p_{1},\;p_{2},\ldots p_{m}\right\}$. Let X be the evaluation indicator set, denoted as $X=\left\{x_{1},\;x_{2},\ldots x_{n}\right\}$. Given the varying levels of knowledge and authority among the experts, the weight of each expert is defined as $\left\{\lambda_{1},\;\lambda_{2},\ldots \lambda_{n}\right\}$. Each expert is asked to select S indicators that they consider important, defining the indicator set chosen by the kth expert as $X^{k}=\left\{{x_{1}}^{k},\;{x_{2}}^{k},\ldots {x_{S}}^{k}\right\}$. At this point, the evaluation indicator matrix can be established as follows:

$${\left[x_{ij}\right]}_{1\times n}=\left[\begin{array}{lllll} x_{11} & x_{12} & x_{13} & \ldots & x_{1n}\\ x_{21} & x_{22} & x_{23} & \ldots & x_{2n}\\ x_{31} & x_{32} & x_{33} & \ldots & x_{3n}\\ \vdots & \vdots & \vdots & \ldots & x_{4n}\\ x_{s1} & x_{s2} & x_{s3} & \ldots & x_{sn} \end{array}\right]$$

To count the occurrences of each indicator, we defined the following: if the jth evaluation indicator $X_{j}$ selected by the kth expert belonged to the indicator set $X^{k}$, it was denoted as $\mu_{k}\left(j\right)=1$; if the indicator did not belong to the indicator set $X^{k}$, it was denoted as $\mu_{k}\left(j\right)=0$. This can be expressed as a function according to Equation (14):

(14) $$\mu_{k}\left(j\right)=\frac{1,If\ldots x_{j}\in X^{k}}{0,If\ldots x_{j}\notin X^{k}}$$

Due to the differing weight coefficients of the experts, the following is true:

(15) $$g_{i}=\sum_{k=1}^{1}\lambda_{k}\mu_{k}\left(j\right)$$

The evaluation indicator matrix provided by experts with different weights can be derived as follows:

$$\left[g_{i}\right]={\left[\lambda \right]}_{1\times s}\times \left[x_{ij}\right]=\left[\lambda_{1},\lambda_{2},\lambda_{3},\ldots \lambda_{S}\right]\times \left[x_{ij}\right]$$

(16) $${\left[x_{ij}\right]}_{1\times n}=\left[\begin{array}{lllll} x_{11} & x_{12} & x_{13} & \ldots & x_{1n}\\ x_{21} & x_{22} & x_{23} & \ldots & x_{2n}\\ x_{31} & x_{32} & x_{33} & \ldots & x_{3n}\\ \vdots & \vdots & \vdots & \ldots & x_{4n}\\ x_{s1} & x_{s2} & x_{s3} & \ldots & x_{sn} \end{array}\right]$$

The weight of the jth indicator is calculated as follows:

(17) $$\omega_{j}=\frac{g_{j}}{\sum_{j=1}^{n}g_{j}}$$

#### 2.4.3. Combination Weighting Method

In the above calculations, this study employed the Delphi method to assign subjective weights to the various performance indicators of CGMs, while the entropy weight method was used to derive the objective weights. However, relying solely on either subjective or objective weights does not provide a complete or accurate reflection of the true significance of the indicators. Therefore, to integrate both perspectives, this study applied the Lagrange multiplier method to combine the subjective and objective weights in a rational manner, resulting in a comprehensive weight W. The equation for this is expressed as follows:

(18) $$W=\frac{\sqrt{W_{j}^{\prime }W_{j}^{\prime \prime }}}{\sum_{j=1}^{n}\sqrt{W_{j}^{\prime }W_{j}^{\prime \prime }}}$$

where W represents the combined weight of the *j*th evaluation index; $W_{j}^{\prime }$ is the *j*th evaluation index derived from the entropy weight method; and $W_{j}^{\prime \prime }$ is the weight of the *j*th evaluation index determined using the Delphi method.

#### 2.4.4. TOPSIS Model

To construct a decision matrix, assume a multi-attribute decision-making problem with m alternative solutions $B=\left\{B_{1},\;B_{2},\ldots B_{m}\right\}$ and n decision attribute indicators $B=\left\{D_{1},\;D_{2},\ldots D_{n}\right\}$. The evaluation values constitute the decision matrix $E={\left(e_{ij}\right)}_{m\times n}$. The specific steps are as follows:

(1) Standardization of the decision matrix.

The original data series is dimensionlessly normalized as follows:

(19) $$f_{ij}=\frac{e_{ij}-\min\left(e_{1j},e_{2j},\ldots ,e_{mj}\right)}{\max\left(e_{1j},e_{2j},\ldots ,e_{mj}\right)-\min\left(e_{1j},e_{2j},\ldots ,e_{mj}\right)}$$

The resulting dimensionless matrix is represented as follows:

(20) $$F={\left(f_{ij}\right)}_{m\times n}$$

(2) Calculation of the weighted normalized decision matrix.

The values of the weighted decision matrix are calculated as follows:

(21) $$H_{ij}=F_{i}\times \omega_{j}$$

In this equation, $\omega_{j}$ is the weight of $D_{j}$ and $\sum_{j=1}^{n}\omega =1$.

(3) Determination of the positive ideal solution and the negative ideal solution.

The positive ideal solution $H^{+}$ is composed of the maximum values from each column in H, while the negative ideal solution $H^{-}$ is composed of the minimum values from each column in H as follows:

(22) $$H^{+}=\left\{\max H_{i1},\max H_{i2},\ldots ,\max H_{in}\right\}$$

(23) $$H^{-}=\left\{\min H_{i1},\min H_{i2},\ldots ,\min H_{in}\right\}$$

(4) Calculation of the distance from each alternative to the positive ideal point $S_{i}^{+}$ and the distance to the negative ideal point $S_{i}^{-}$. The distances are calculated as follows:

(24) $$S_{i}^{+}=\sqrt{{\sum_{j=1}^{n}\left(\max H_{ij}-H_{ij}\right)}^{2}}$$

(25) $$S_{i}^{-}=\sqrt{{\sum_{j=1}^{n}\left(\min H_{ij}-H_{ij}\right)}^{2}}$$

(5) Calculation of the relative closeness of alternative solutions to the positive ideal solution. The relative closeness $G_{i}$ of the alternative solutions to the positive ideal solution is calculated as follows:

(26) $$G_{i}=\frac{S_{i}^{-}}{S_{i}^{+}+S_{i}^{-}}$$

where 0 ≤ $G_{i}$ ≤ 1; as $G_{i}$ approaches 1, it indicates that the evaluation object is close to the positive ideal solution, while as $G_{i}$ approaches 0, it signifies that the evaluation object is close to the negative ideal solution.

## 3. Results and Discussions

### 3.1. Mechanical Properties Analysis

**Figure 8:**
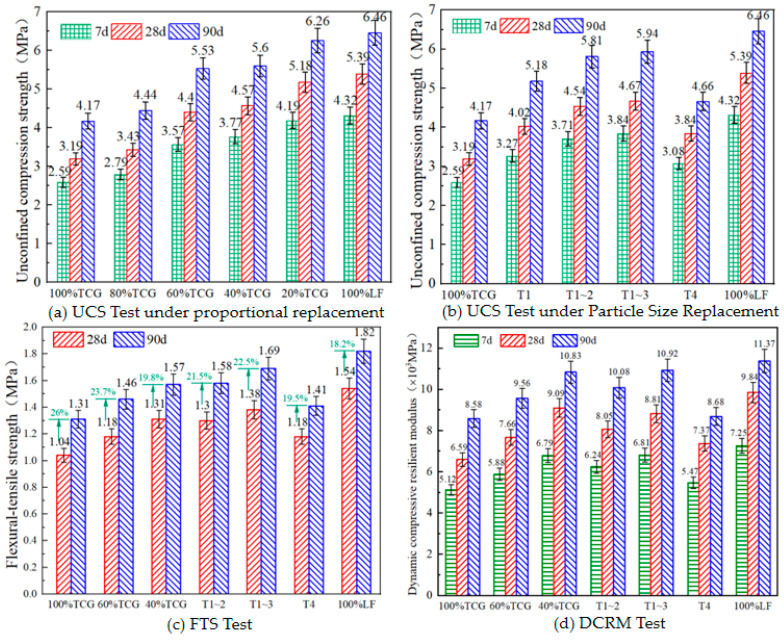
Test results of UCS, FTS, DCRM.

To investigate the mechanical properties of different coal gangue mixtures, we conducted tests of strength properties such as UCS, FTS, and DCRM. The research results are shown in Figure 8.

The unconfined compressive strength (UCS) of the mixtures under different replacement strategies at 7, 28, and 90 days is presented in Figure 8a,b. The findings show that gravel substitution significantly increased mixture UCS: gravel-replaced mixtures exhibited 7.7%, 37.8%, 45.6%, and 61.8% UCS increases vs. 100% TCG, attributed to gravel’s superior compressive properties enhancing aggregate strength. Conversely, coal gangue’s high crushing value and irregular shape reduced inter-particle friction and mechanical interlocking, leading to lower UCS in high-gangue mixtures [43]. Notably, 80% TCG showed only a 7.7% UCS increase over 100% TCG, while 100% LF increased by 5% vs. 20% TCG, indicating minimal UCS changes with 80% or 20% TCG replacement. Based on this, a 40–60% coal gangue content is recommended for optimal performance and resource efficiency.

The UCS growth rates of the CGMs changed over time: 100% LF showed the highest 7–28 d increase, with 100% TCG also rising significantly; other mixtures exhibited >21% rates with minor differences. From 28 d to 90 d, strength growth declined with increasing gravel content: 100% TCG grew by 30.7% vs. 19.8% for 100% LF due to weakened cement hydration and gradually enhanced pozzolanic reactions [44]. In size-replaced mixtures, UCS growth rates ranked T1 > T1~2 > T1~3, indicating coarse coal gangue aggregates promote long-term strength via their microporous structure, which retains and slowly releases moisture for internal curing [45].

FTS tests on seven CGM types (Figure 8c) showed that gravel improved FTS, while blending methods impacted interfacial bonding and transition zone characteristics, leading to varied FTS trends. Under proportional replacement, FTS followed 100% TCG < 60% TCG < 40% TCG < 100% LF, as coal gangue inhibited cement hydration, weakening binder FTS. For size-replaced mixtures, the order was 100% TCG < T4 < T1~2 < T1~3 <100% LF. T4’s 19.5% FTS increase over 100% TCG contrasted with the latter’s 26% rise, indicating that late-stage pozzolanic reactions enhance bonding despite fine gangue’s negative effect. T1~2 and T1~3 showed similar FTS, suggesting comparable strength contributions.

DCRM tests for all the mixtures at 7 d, 28 d, and 90 d (Figure 8d) showed that increased gravel content enhanced aggregate interlocking, improving primary skeleton stiffness. Coal gangue promoted cement hydration, raising DCRM. Higher 7 d DCRM correlated with greater later-stage compressive DCRM, though growth rates variev: 100% TCG’s modulus increased by 28.6% (7 d–28 d) and 67.6% (7 d–90 d), 40% TCG rose by 33.8% and 59.4%, while T4 increased by 31.8% and 58.7% (lower than 100% TCG’s 90 d growth). This indicates that coal gangue positively impacted DCRM via enhanced bonding and void filling from pozzolanic reactions. Under rational blending, the CGMs’ DCRM values were lower than those of 100% LF, signaling improved mechanical performance and flexibility for better deformation resistance under load. Practically, coal gangue enhanced mixture flexibility and DCRM growth potential, expanding application scenarios.

Based on comprehensive analysis, test results showed that coal gangue content correlated with reduced mechanical performance: 100% TCG’s 28 d UCS was 59.2% of pure limestone, reflecting its porous structure and low strength weakening the matrix’s load-bearing. Each 20% increase cut UCS by 18.7%, with steeper FTS decline due to weak interfacial zones. DCRM curves (Figure 8c) showed a nonlinear rebound modulus decay under cyclic loading, accelerating beyond 60% gangue (42% steeper slope), indicating particle crushing harmed long-term stiffness. Coarse aggregate replacement (T1~3) improved properties via gradation: 90 d UCS reached 4.67 MPa (46.4% over TCG), with DCRM growing by 12.3% after 28 d. This synergy between the coarse aggregate skeleton support and coal gangue’s microporous internal curing (slow moisture release for late hydration) provides a basis for gradation design.

### 3.2. Durability Analysis

**Figure 9:**
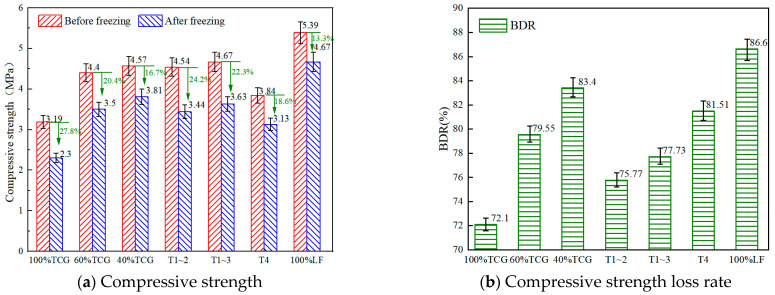
Test results of the freeze–thaw cycles.

**Figure 10:**
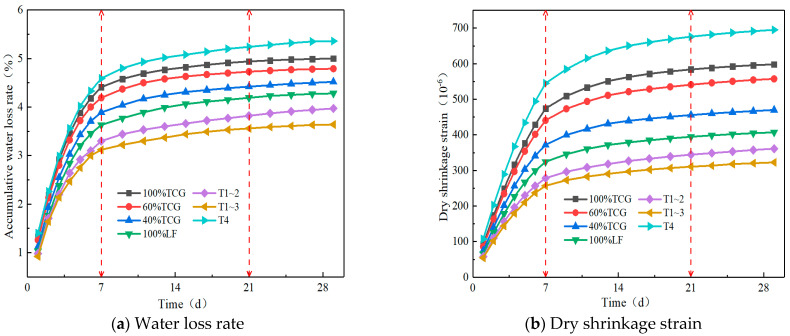
Shrinkage test results.

To investigate the durability properties of different coal gangue mixtures, we conducted tests of durability characteristics such as freeze–thaw cycles, dry shrinkage, and fatigue. The research results are shown in Figure 9 and Figure 10 below.

(1) Frost resistance and drying shrinkage

Freeze–thaw tests on seven CGM types (Figure 9) evaluated frost resistance, showing that the residual strength decreased with a higher coal gangue content, leading to greater strength loss and lower frost resistance. This stemmed from coal gangue’s higher porosity, irregular bonding surfaces increasing voids/absorption, and lower compressive strength, making it prone to damage during cycles, intensifying pore-related strength loss [46]. Coarse/fine aggregate replacement improved the residual strength ratios: T1~3 and T1~2 showed minimal differences, indicating that grade 3 aggregates had a limited frost resistance impact. T4 exhibited the highest ratio, effectively mitigating freeze–thaw effects; it had a significantly lower strength loss than 100% TCG, with a similar trend between T1~3 and 100% LF.

These results show that fine coal gangue aggregates more significantly affected the mixtures’ frost resistance, likely due to higher water absorption and hindered cement hydration. Replacing fine aggregates in CGMs is a practical approach to enhance frost resistance and reduce the freeze–thaw strength loss.

Figure 10 shows water loss rates and drying shrinkage strains of seven CGM types. In Figure 10a, cumulative water loss rose over time until stabilization; proportional replacement with more gravel reduced the loss due to coal gangue’s porous, low-water-retention structure. For coarse/fine aggregate replacement, water loss rates were as follows: T1~3 < T1~2 < 100% LF < 100% TCG < T4. Coarse aggregate replacement lowered loss (gravel density mitigates evaporation), while fine replacement increased it (fine gangue retains some water but reduces reaction availability, leading to T4’s highest rate).

Figure 10b shows drying shrinkage strain changes over time for the CGMs, with strain trends matching water loss rates; higher loss correlated with greater strain. Under proportional replacement, increasing limestone reduced shrinkage due to coal gangue’s high porosity, water absorption, weak interfacial bonding, and low-strength aggregates’ limited restraint. For size replacement, T1~2 and T1~3 exhibited the smallest shrinkage (smaller than that of 100% LF), while T4 showed the largest (larger than that of 100% TCG), indicating that fine coal gangue aggregates increased mixture shrinkage. Practically, including moderate fine gangue and ensuring 7-day curing water retention can mitigate drying shrinkage.

Comprehensive analysis showed that the freeze–thaw and drying shrinkage tests (Figure 9 and Figure 10) revealed coal gangue mixtures’ multi-scale failure mechanisms: the freeze–thaw residual strength ratio (BDR) negatively correlated with gangue content, dropping 6.5% per each 20% increase. SEM analysis showed that high-content TCG had unreacted gypsum crystals (peak strength decreased by 23%) and loose C–S–H gel in ITZ, increasing water paths and frost stress. XRD indicated that T1~3’s coarse gradation lowered porosity by 14.8% and boosted ITZ Aft crystals to 37.5%, mitigating the freeze–thaw damage.

(2) Fatigue property

**Figure 11:**
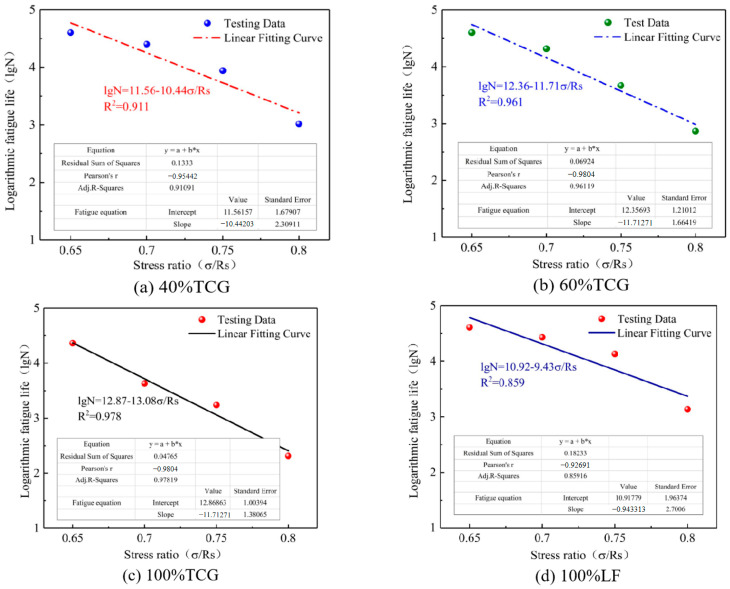

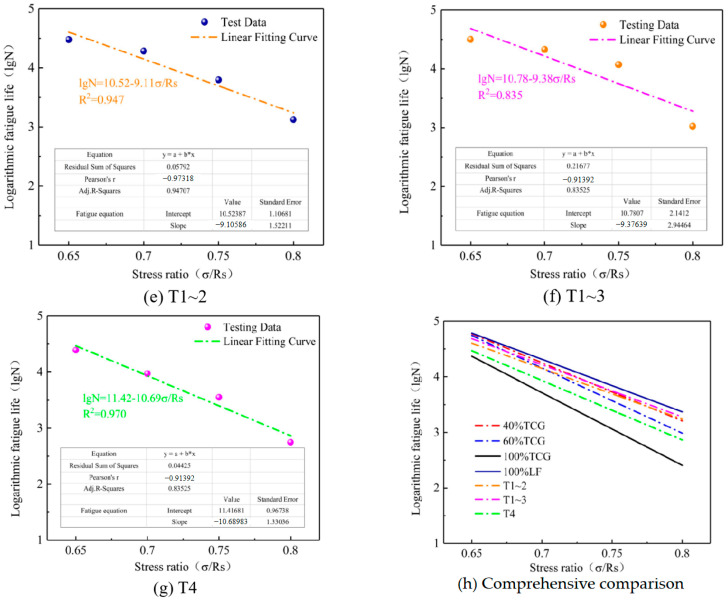
Fatigue curves.

Based on the FTS results, stress levels were set at 0.65, 0.7, 0.75, and 0.8. Four-point bending fatigue tests were conducted to obtain the fatigue life of the mixtures at the corresponding stress ratios. After taking the logarithm of the results, the fatigue life prediction curves and equations for each mixture were fitted according to Equation (27), as shown in Figure 11.

(27) $$\mathrm{lg}N=A+B\left(\sigma /Rs\right)$$

where lg⁡N represents the logarithmic fatigue life, σ/Rs denotes the stress ratio level, and A and B are the intercept and slope of the fatigue life prediction curve, respectively.

As shown in Figure 11, the fatigue life of the CGMs significantly improved with the incorporation of limestone. This can be attributed to the higher water absorption of coal gangue and the presence of impurities, such as coal dust, which negatively affect the effective water-to-binder ratio in the surrounding cement paste. As a result, the formation of C–S–H gel and Aft crystals around the aggregates was hindered, leading to a weak interfacial transition zone (ITZ). When the proportion of coal gangue was elevated, the number of weak ITZs increased, diminishing the mixture’s ability to withstand cyclic fatigue loading.

Under proportional replacement, the fatigue life ranking of the mixtures was as follows: 100% TCG < 60% TCG < 40% TCG < 100% LF. The fatigue life differences between the mixtures were notably influenced by the stress ratio. At low stress ratios, the differences in fatigue life between 60% TCG, 40% TCG, and 100% LF were minimal. However, as the stress ratio increased, these differences became more pronounced, indicating that proportional replacement with limestone was particularly advantageous under low-stress conditions. For size replacement, the fatigue life ranking was as follows: 100% TCG < T4 < T1~2 < T1~3 < 100% LF. Notably, the fatigue life difference of T4, compared to other mixtures, increased as the stress ratio rose, suggesting a greater sensitivity to stress ratio in size replacement mixtures. The mixtures exhibited varying performance across different stress levels: at low stress levels, 40% TCG and 60% TCG demonstrated superior fatigue life, while T1~3 and T1~2 performed less effectively. Conversely, at high stress levels, T1~3 and T1~2 outperformed 40% TCG and 60% TCG in fatigue life. These findings highlight the differential effects of blending methods on the fatigue life of CGMs. Proportional replacement mixtures excel under low stress ratios, while size replacement mixtures are better suited for high stress ratio scenarios. This distinction underscores the importance of selecting appropriate blending strategies based on the anticipated stress conditions to optimize fatigue performance.

### 3.3. Comprehensive Performance Evaluation and Analysis

(1) Economic indicator

Based on market material prices, Table 7 lists the unit prices of the seven mixtures. The unit prices for coal gangue and limestone are 35 RMB/t and 110 RMB/t, respectively. Using a base of 1 t of material, a cost analysis was performed according to the determined proportions (1#:2#:3#:4# = 27:25:20:28).

(2) Environmental indicator

The total energy consumption (EC) of each mixture during production, transportation, and construction was calculated using the quota method, as shown in Table 8. The quota method relies on current budget estimates, calculating the number of construction machines and the fuel consumption of each unit of equipment, and then multiplying these factors to determine the total fuel consumption for the entire process. While calculating the EC, it is essential to consider its net calorific value (NCV), which is the amount of heat produced when a fuel is completely burned within a specific mass or volume. The calculation formula is as follows:

(28) $$E_{i}=F_{i}\times NCV_{i}$$

where $E_{i}$ represents the EC of a particular fuel; $F_{i}$ denotes the quantity of that fuel consumed; and $NCV_{i}$ refers to the net calorific value of the fuel.

(3) DET evaluation results

Although the mechanical and durability properties of the CGMs with different blending schemes had been evaluated, a comprehensive quantitative comparison of the overall performance of these mixtures at the same level had not yet been conducted. To address this, ADET was employed to assess the overall performance of the CGMs. The evaluation indicators for the mixtures are summarized in Table 9. The standardization matrix of evaluation indexes for all the mixtures processed using Equation (8) is listed in Table 10.

Table 11 summarizes the calculation results for the subjective and objective weights of the evaluation indicators, determined using the Delphi method and the entropy weight method. These weights were derived using Equations (9)–(13) for the Delphi method and Equations (14)–(17) for the entropy weight method. Additionally, the Delphi–entropy combined weights, calculated using Equation (18), are also presented in Table 11.

With the different weights presented in Table 11, this study adopted an improved TOPSIS model to calculate the scores of the seven kinds of mixtures. We input the weights obtained using the three different methods in Table 11 into the TOPSIS comprehensive evaluation method to calculate the ranking scores shown in Table 12. The overall performance rankings of the seven mixtures are presented in Table 12.

From Table 12, the final evaluation results of the overall performance of the CGMs using the combined weighting method based on subjective and objective assessments (TOPSIS theory) indicate that T1~3 was the optimal mixture, followed by 100% LF and T1~2. Conversely, 100% TCG and T4 exhibited the poorest overall performance, which correlated with the superior mechanical strength and durability demonstrated by the T1~3 blending scheme. This finding also partially validates the weight distribution of various performance indicators determined through the Delphi method and the entropy weight method, supporting the reliability of the comprehensive performance evaluation model for CGMs established using the TOPSIS method.

The advanced radar chart method was used to quantitatively compare the scores of the comprehensive performance of the coal mixture obtained by the DET. The evaluation index matrix was established using Equation (29), and the nonlinear equation transformation was carried out using Equation (30).

(29) $$b_{ij}=\frac{a_{ij}-E\left(y_{j}\right)}{\sigma \left(y_{i}\right)}$$

(30) $$r_{ij}=\frac{2}{\pi }\arctan\left(b_{ij}\right)+1$$

where $b_{ij}$ represents each indicator after standardization, $E\left(y_{j}\right)$ is the average of indicator j, $\sigma \left(y_{i}\right)$ is the standard deviation of indicator i, and $r_{ij}$ represents the j evaluation index of the ith evaluation object after the basic indicator’s nonlinear transformation. Referring to Equations (29) and (30), $A_{i}=\left[b_{ij},r_{ij}\right]$ was calculated as follows:

$$A_{1}=\left[0.3503,1.2145\right];A_{2}=\left[-0.7505,0.5901\right];$$

$$A_{3}=\left[0.6593,1.3711\right];A_{4}=\left[1.2694,1.5752\right];$$

$$A_{5}=\left[-1.4295,0.3886\right];A_{6}=\left[-0.8305,0.5588\right];$$

$$A_{7}=\left[0.7314,1.4020\right].$$

**Figure 12:**
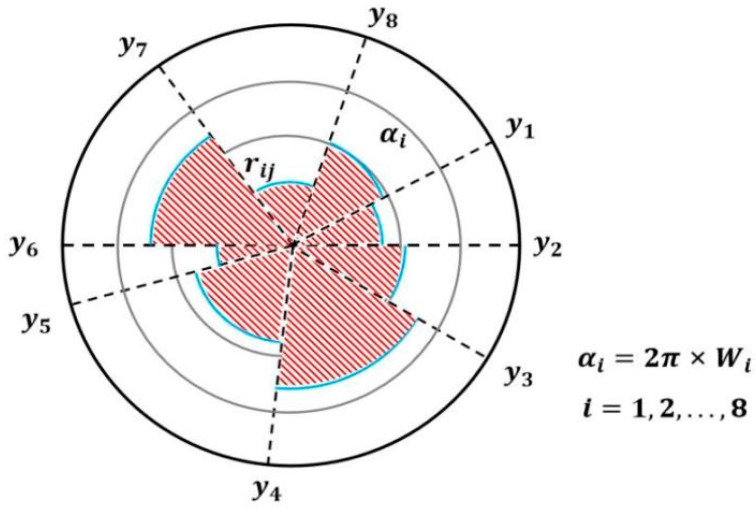
Sketch map of the radar chart evaluation method.

The sector angle and the radius are two critical characteristics used to draw a radar chart. As illustrated in Figure 12, the sector angle $\alpha_{i}$ is determined using the Delphi–entropy combination weights $W_{i}$ to represent the importance degree of the evaluation indexes, which is calculated as follows:

(31) $${\alpha_{i}=2\pi \times W}_{i},\;i=1,2,\ldots ,8$$

**Figure 13:**
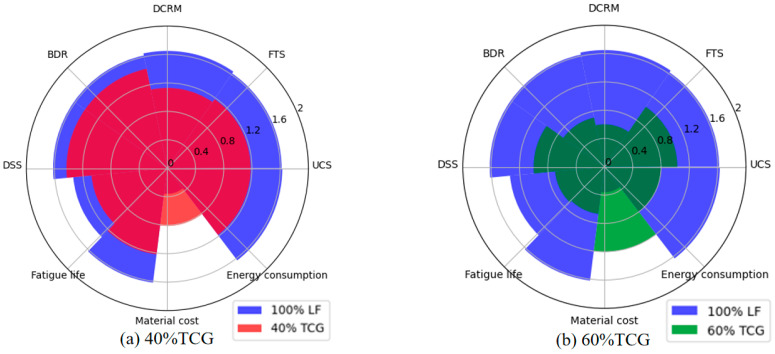

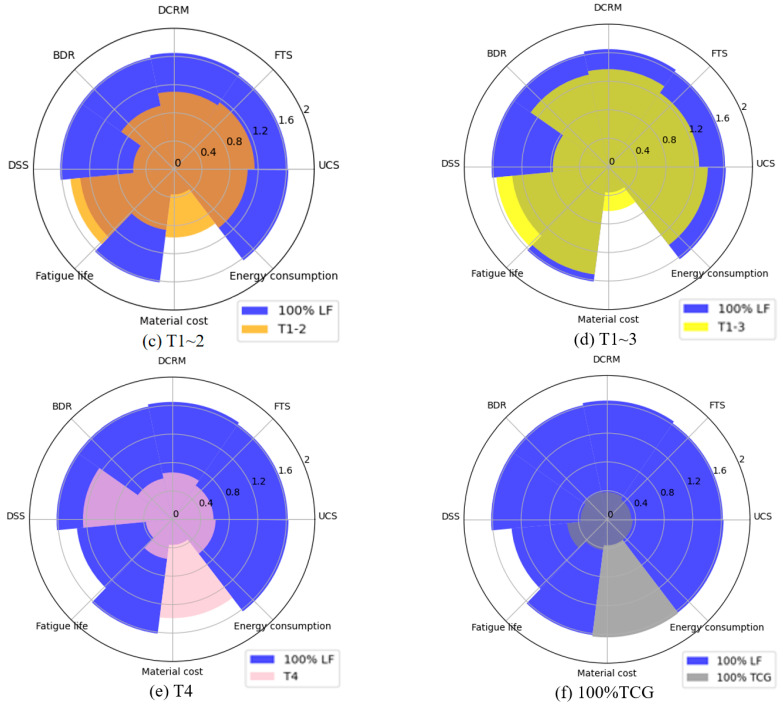
Comparison of the comprehensive performance of each group of mixtures with 100% LF.

The radius of each sector was determined based on the evaluation object following a nonlinear transformation of the basic indicators. The radar charts in Figure 13 compare the comprehensive evaluation indices of different mixtures against the 100% LF baseline.

### 3.4. Microscopic Mechanism Analysis

(1) XRD Analysis

**Figure 14:**
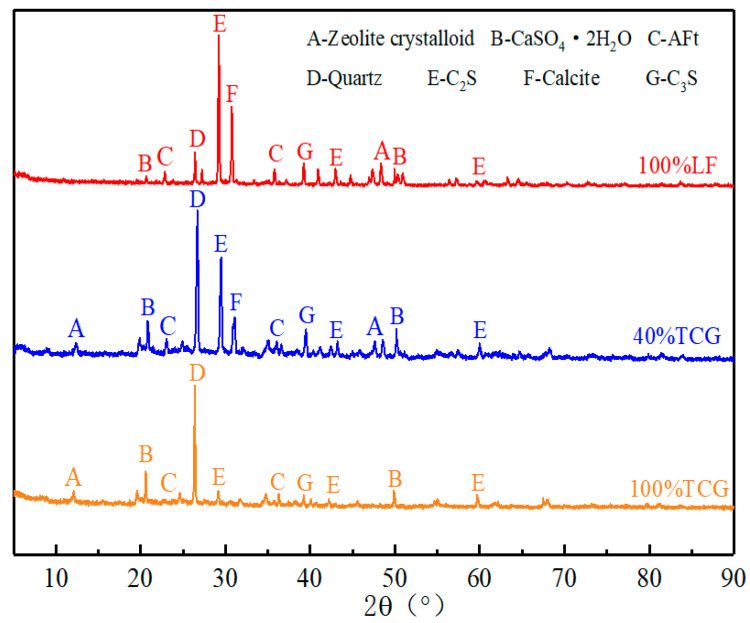
XRD test results.

Figure 14 presents the XRD test results for 100% LF, 100% TCG, and 40% TCG. The mineral composition of the samples was complex, with the main characteristic peaks corresponding to dicalcium silicate (C_2_S), tricalcium silicate (C_3_S), quartz (SiO_2_), and gypsum (CaSO_4_·2H_2_O). A higher peak indicates a greater content of the respective material. Variations in the C_2_S and C_3_S peak heights across the XRD spectra of 40% TCG, 100% TCG, and 100% LF reflect differences in the hydration rates and degrees of hydration between the three mixtures.

The spectra also revealed the presence of unreacted gypsum in all three mixtures, which, due to its inherently low strength, diminishes the overall strength of the mixtures [37]. Notably, the characteristic peaks of gypsum were more prominent in 100% TCG and 40% TCG, contributing to their lower strength compared to 100% LF. Additionally, the spectra indicated the presence of Aft crystals and zeolite-like crystals, which played a beneficial role in the strength development of the mixtures. The content of these strength-enhancing crystals was highest in 100% LF and lowest in 100% TCG, suggesting that coal gangue aggregates impeded gel formation, thereby reducing the overall strength of the mixtures.

(2) SEM Analysis

To analyze the hydration mechanism of the cemented gangue mixtures (CGMs) and provide support for the research on their mechanical properties and durability, we conducted SEM tests on six different gradation types of CGMs. The test results are as follows.

**Figure 15:**
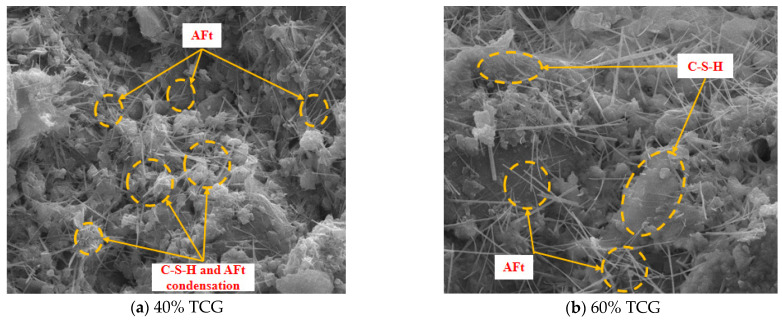

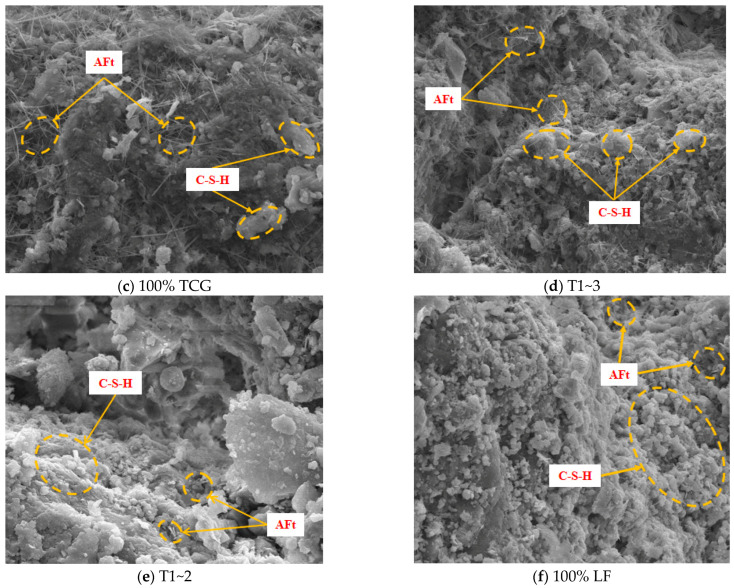
SEM test results.

Figure 15 shows SEM results for the CGMs, with bonding characteristics varying by aggregate proportion. Images (a)–(d) reveal that 40% TCG, 60% TCG, 100% TCG, and T1~3 all contained C–S–H gel and Aft crystals, indicating hydration. Comparing (a) and (b), 40% and 60% TCG showed sufficient hydration with Aft and C–S–H: 60% TCG had scattered Aft and sparse gel, while 40% TCG featured large C–S–H areas intertwined with Aft, suggesting stronger cohesion and bonding potential.

Figure 15c,d shows that T1~3 had more AFt crystals than 100% TCG, with some bridging pores and a denser C–S–H gel network enhancing material density. T1~2 (Figure 15e) resembled 100% TCG with AFt and C–S–H but with shorter crystals and less bonding material. In 100% LF (Figure 15f), C–S–H gel around AFt developed rapidly, filling voids from needle-like AFt to form cloud-like clusters, matching prior XRD results and improving structural density and inter-aggregate cohesion. Macroscopically, this translated to the mixture’s superior strength, fatigue, and shrinkage performance.

Figure 15’s SEM–EDS surface scanning quantified ITZ elemental distribution: T1~3 had a Ca/Si ratio of 2.15 (vs. 1.62 for 100% TCG), indicating more C–S–H gel in hydration products. XRD showed that T1~3’s C–S–H content was 43.7% (vs. 29.4% for TCG). AFt crystal aspect ratio correlated positively with UCS, revealing crystal morphology’s role in macroscopic strength.

## 4. Conclusions

To improve the utilization of coal gangue solid waste in road engineering and enhance its value for high-value applications, this study investigated the preparation of CGMs using two blending approaches: proportional replacement and particle size replacement. The study evaluated the mechanical strength and durability of these mixtures, and employed microscopic techniques to examine the effects of different blending strategies on road performance, particularly the distinct roles of coarse and fine aggregates. Additionally, a DET evaluation model was proposed, integrating both subjective and objective weighting methods to assess the importance of various indicators and establish a comprehensive performance evaluation framework for CGMs. The main conclusions drawn from this study are as follows:

(1) The density of coal gangue is comparable to that of gravel, and it exhibits favorable particle gradation. Coal gangue primarily consists of minerals such as quartz, kaolinite, and illite, with a flaky or layered internal structure and some voids. This results in a relatively loose arrangement. The physical and chemical properties of coal gangue satisfy the application requirements for expressway subbase layers under heavy, medium, and light traffic conditions.
(2) As the proportion of coal gangue in the mixture increases, there is a general decrease in UCS, DCRM, FTS, BDR, and fatigue life, while DSS shows an increase. However, over time, particularly with increased curing age, mechanical properties improve. Specifically, the UCS increased by 25.6%, 22.5%, and 19.8% for 60% TCG, 40% TCG, and 100% LF mixtures, respectively, between 28 and 90 days of curing. Both blending methods—proportional and particle size replacement—lead to enhanced fatigue life, with proportional replacement demonstrating better fatigue performance at lower stress ratios. In contrast, particle size replacement notably improves the stress sensitivity of the mixtures, making them more suitable for higher stress ratio conditions.
(3) Coarse coal gangue aggregates have minimal impact on the freeze–thaw resistance of mixtures but significantly weaken the drying shrinkage performance. Replacing coal gangue aggregates of sizes 19~31.5 mm and 9.5~19 mm can significantly enhance the UCS, FTS, and DCRM of the mixtures, while the enhancement effect of replacing aggregates sized 4.75~9.5 mm is not obvious. Fine coal gangue aggregates inhibit early hydration reactions and have a considerable negative impact on freeze–thaw resistance but can benefit the improvement of mechanical performance over time and help mitigate drying shrinkage.
(4) Hydration products of coal–gravel mixtures at 7 d mainly include AFt crystals and zeolite-type crystals, with unreacted C_2_S and C_3_S also present. Mixtures with 40% TCG and T1~3 exhibit large areas of C–S–H gel and AFt crystals forming a cohesive gel network, further enhancing the material’s density. A higher proportion of coal gangue leads to a greater quantity of fine aggregates, which slows the hydration reaction.
(5) The evaluation indicators of the DET model for CGMs rank as follows: T1~3 > 100% LF > T1~2 > 40% TCG > 60% TCG > 100% TCG > T4. The comprehensive performance evaluation identified T1~3 as the most favorable mixture, making it the recommended optimal blending scheme for express highway subbases.

This study examined the mechanical strength and durability of CGMs and investigated the mechanisms of internal strength formation and failure using microscopic analysis. Future research will aim to conduct both qualitative and quantitative XRD analyses, delve deeper into the mechanisms of internal strength formation through nanoindentation tests and differential scanning calorimetry, and explore the interactions between coal gangue and limestone in greater detail.

## Figures and Tables

**Table 1:** Technical parameters of coal gangue.

Index	Material Specification			Standard	Testing Standard	Testing Method
	19~31.5 mm	9.5~19 mm	4.75~9.5 mm			
Apparent density (g/cm^3^)	2.578	2.573	2.572	—	JTG/T E42-2005 [33]	T 0304-2005 [33] Wire Basket Method
Water absorption (%)	1.68	2.14	2.66	—	JTG/T E42-2005	T 0304-2005 Wire Basket Method
Needle sheet content (%)	15.6	10.3	13.8	—	JTG/T E42-2005	T 0312-2022 [33] Vernier Caliper Method
Disintegration resistance index (%)	99.1			—	JTG E51-2009 [34]	T 0843-2009 [34] Unconfined Compressive Strength Test
Crushing value (%)	23.7			≤26	JTG F20-2015 [35]	T 0316-2005 [35] Crushed Value Test for Coarse Aggregates
Loss on ignition (%)	8.9			≤10	GB/T 176-2011 [36]	GB/T 176-2011 [36] Loss on Ignition Test
Liquid and plastic limit (%)	11.6			≤17	ASTM D4318 [37]	T 0118-2007 [37] Combined Determination of Liquid Limit and Plastic Limit

**Table 2:** Chemical composition of coal gangue.

Oxide Type	SiO_2_	Al_2_O_3_	CaO	Fe_2_O_3_	K_2_O	MgO
Content (%)	47.02	21.37	5.85	4.00	2.38	1.03

**Table 3:** Technical parameters of limestone.

Index	Material Specification			Standard	Testing Standard	Testing Method
	19~31.5 mm	9.5~19 mm	4.75~9.5 mm			
Apparent density (g/cm^3^)	2.684	2.650	2.632	—	JTG/T E42-2005	T 0304-2005 Wire Basket Method
Water absorption (%)	0.46	0.72	0.97	—	JTG/T E42-2005	T 0304-2005 Wire Basket Method
Needle sheet content (%)	5.4	7.2	6.2	—	JTG/T E42-2005	T 0312-2022 Vernier Caliper Method
Crushing value (%)	21.2			≤26	JTG F20-2015	T 0316-2005 Crushed Value Test for Coarse Aggregates

**Table 4:** Technical parameters of cement.

Test Items		Test Results	Standard	Test Method
Fineness (%)		6.7	≤10	T0502-2005
Initial setting time (min)		210	≥180	T0505-2020
Final setting time (min)		480	360~600	T0505-2020
Stability (mm)		1.5	≤5.0	T0505-2020
Flexural strength (MPa)	7 d	4.8	≥3.5	T0506-2005
	28 d	8.2	≥6.5	
Compressive strength (MPa)	7 d	24.3	≥17	T0506-2005
	28 d	50.5	≥42.5	

**Table 5:** The pass rate of different screen apertures.

Sieve Size (mm)	Range of Grading	Median	Composite Gradation
31.5	100~100	100.0	100
19	68~86	77.0	74.7
9.5	38~58	48.0	48.9
4.75	22~32	27.0	28.4
2.36	16~28	22.0	19.7
0.6	8~15	11.5	9.4
0.075	0~3	1.5	1.7

**Table 6:** Experimental blending schemes for coal gangue and limestone.

Mixing Type	Replace Method				Number
	19~31.5 mm	9.5~19 mm	4.75~9.5 mm	0~4.75 mm	
Particle size replacement	LF	TCG	TCG	TCG	T1
	LF	LF	TCG	TCG	T1~2
	LF	LF	LF	TCG	T1~3
	TCG	TCG	TCG	LF	T4
Proportional replacement	80% TCG + 20% LF				80% TCG
	60% TCG + 40% LF				60% TCG
	40% TCG + 60% LF				40% TCG
	20% TCG + 80% LF				20% TCG
Control group	100% TCG				100% TCG
	100% LF				100% LF

**Table 7:** Price list of the seven kinds of mixtures.

Type of Mixture	Proportion of Coal Gangue (%)	Percentage of Limestone (%)	Total Price (RMB/t)
40% TCG	40	60	80
60% TCG	60	40	65
100% TCG	100	0	74
100% LF	0	100	89
T1~2	52	48	56
T1~3	28	72	35
T4	72	28	110

**Table 8:** EC table of the seven kinds of mixtures.

Energy Consumption Indicators	40% TCG	60% TCG	100% TCG	100% LF	T1~2	T1~3	T4	AVG	STDEV
Raw material production	32.668	32.542	32.29	32.92	32.59	32.74	32.47	32.6	0.2
Raw material transportation	23.39	23.39	23.39	23.39	23.39	23.39	23.39	23.39	0
Mixture production	309.308	311.712	316.52	304.5	310.75	307.87	313.16	310.55	3.86
Mixture transportation	9.04	9.11	9.25	8.9	9.082	9	8.9	9.04	0.12
Construction phase	61.232	60.628	59.42	62.44	60.87	61.6	60.26	60.92	0.97
Total EC (MJ/t)	435.638	437.382	440.87	432.15	436.182	434.59	438.432	436.46	2.8

**Table 9:** Summary of evaluation indexes of the seven kinds of mixtures.

Performance	40% TCG	60% TCG	T1~2	T1~3	T4	100% TCG	100% LF	AVG	STDEV
UCS (Mpa)	4.57	4.40	4.54	4.67	3.84	3.19	5.39	4.37	0.69
FTS (Mpa)	1.31	1.18	1.30	1.38	1.18	1.04	1.54	1.28	0.16
DCRM (Mpa)	9092	7657	8053	8813	7368	6590	9840	8201.86	1115.18
BDR (%)	83.40	79.55	75.77	77.73	81.51	72.10	86.64	79.53	4.87
DSS (10^−6^)	470.1	557.5	361.2	322.6	695.3	598.2	407.2	212.86	135.54
Fatigue life (cycles)	8746	4690	6282	11834	3579	1743	13577	7207.29	4369.65
Material cost	80	65	74	89	56	35	110	42.29	24.05
Total EC (MJ/t)	374.42	376.75	375.2	373	378.1	381.45	369.71	10.92	3.76

**Table 10:** Standardization matrix of evaluation indexes of the seven kinds of mixtures.

Performance	40% TCG	60% TCG	T1~2	T1~3	T4	100% TCG	100% LF	AVG	STDEV
UCS (Mpa)	0.29	0.04	0.24	0.43	−0.77	−1.71	1.47	1	0
FTS (Mpa)	0.21	−0.59	0.15	0.65	−0.59	−1.46	1.64	1	0
DCRM Mpa)	0.80	−0.49	−0.13	0.55	−0.75	−1.45	1.47	1	0
BDR (%)	0.80	0.00	−0.77	−0.37	0.41	−1.53	1.46	1	0
DSS (10^−6^)	0.13	−0.52	0.93	1.22	−1.53	−0.82	0.59	1	0
Fatigue life (cycles)	0.35	−0.58	−0.21	1.06	−0.83	−1.25	1.46	1	0
Material cost	−0.30	0.32	−0.05	-0.68	0.69	1.57	−1.55	1	0
Total EC (MJ/t)	0.30	−0.32	0.08	0.67	−0.70	−1.57	1.55	1	0

**Table 11:** Calculation values of weights using three weighting methods.

Weight Values	UCS (Mpa)	FTS (Mpa)	DCRM (Mpa)	BDR (%)	DSS (10^−6^)	Fatigue Life (Cycles)	Material Cost	Total EC (MJ/t)
Delphi method	0.143	0.119	0.110	0.104	0.135	0.113	0.137	0.140
Entropy weight method	0.135	0.135	0.135	0.136	0.104	0.113	0.113	0.129
Delphi–entropy combination method	0.154	0.129	0.119	0.113	0.113	0.102	0.124	0.145

**Table 12:** Comparison of the scores of the seven kinds of mixtures obtained using three weighting methods.

Type of Mixture	DET Scores	Performance Ranking	Type of Mixture
40% TCG	0.53915	The comprehensive performance ranking of the seven kinds of mixtures (from high to low)	T1~3
60% TCG	0.43688		100% LF
T1~2	0.56786		T1~2
T1~3	0.62454		40% TCG
T4	0.37380		60% TCG
100% TCG	0.42945		100% TCG
100% LF	0.57455		T4

## Data Availability

The original contributions presented in this study are included in the article. Further inquiries can be directed to the corresponding author.
